# The association between antibodies to neurotropic pathogens and schizophrenia: a case-control study

**DOI:** 10.1038/npjschz.2015.41

**Published:** 2015-11-04

**Authors:** Lot D de Witte, Hans C van Mierlo, Manja Litjens, Hans C Klein, Sabine Bahn, Ab D Osterhaus, the GROUP Investigators

**Affiliations:** 1 Department of Psychiatry, Brain Center Rudolf Magnus, University Medical Center Utrecht, Utrecht, The Netherlands; 2 Department of Psychiatry, University of Groningen, Groningen, The Netherlands; 3 Department of Chemical Engineering and Biotechnology, University of Cambridge, Cambridge, UK; 4 Department of Viroscience, Erasmus University Medical Center, Rotterdam, The Netherlands

## Abstract

**Background:**

Exposure to neurotropic pathogens has been proposed as an environmental risk factor for schizophrenia and can be evaluated by measuring pathogen-specific immunoglobulin G (IgG). Seroprevalence of pathogen-specific IgG reflects prior exposure, whereas IgG levels are associated with reactivity or reinfection. Several studies have examined these parameters in schizophrenia. However, results still remain inconclusive, as several previous studies did not correct for important confounding factors.

**Aims:**

To investigate whether schizophrenia is associated with prior exposure to neurotropic pathogens, or with their reactivation.

**Methods:**

We examined the seroprevalence and titer of IgG antibodies against herpes simplex virus-1 and -2 (HSV-1/HSV-2), varicella zoster virus (VZV), Epstein–Barr virus (EBV), cytomegalovirus (CMV), and *Toxoplasma gondii* (TG) in plasma of 368 adult patients with a schizophrenia spectrum disorder and 282 controls using ELISA.

**Results:**

We did not find evidence for an increased exposure to HSV-1, HSV-2, EBV, and TG in patients. There was a significantly higher seroprevalence of VZV (98.9% vs. 95.6%, *P*<0.05) and CMV (40.4% vs. 27.7%, *P*<0.001) in controls as compared with patients, which did not remain statistically significant after adjustment for various potential confounders. We did not find significant differences in antibody titers of seropositive patients and controls for any of the six pathogens.

**Conclusions:**

Our results do not support the hypothesis that increased exposure to neurotropic pathogens after birth is associated with schizophrenia.

## Introduction

Although the pathogenesis of schizophrenia is still largely unclear, it is generally accepted that this disease is caused by the interplay between genetic and environmental factors. Microorganisms that can infect the central nervous system (CNS), the so-called neurotropic pathogens, have been proposed as candidate environmental factors for a long time.^1,2^ This hypothesis is supported by the fact that genes associated with schizophrenia are involved in immune processes, including host–pathogen interactions.^3–5^ Other support includes the increased risk for schizophrenia after prenatal^6^ or childhood infections^7^ and the increased prevalence of schizophrenia amongst people born in winter season^8^ and in urban areas.^9^

Specific neurotropic pathogens have been proposed as candidate environmental risk factors for schizophrenia.^1^ These include pathogens that infect a substantial part of the population, such as several types of herpes viruses (herpes simplex virus-1 and -2 (HSV-1/HSV-2), Epstein–Barr virus (EBV), and cytomegalovirus) and the parasite *Toxoplasma gondii* (TG). Another common characteristic of all these pathogens is their ability to cause a latent infection by slowing down their replication and remaining undetected from the immune system by pathogen-specific mechanisms.^10^ These pathogens may therefore affect crucial CNS functions and neurodevelopmental processes during primary infection, but also afterwards.

Prior exposure to a specific pathogen can be determined by analysing immunoglobulin G (IgG) class antibodies. Early during a primary infection immunoglobulin M (IgM) is produced. However, following maturation of B cells, immunoglobulin class switching takes place and IgG is synthesized.^11^ This production generally lasts for the entire lifespan to protect the infected person against reinfection. Measuring pathogen-specific IgG is therefore indicative of exposure to a certain pathogen. An exception is the first 6 months after birth; in this period the new-born still has maternal IgG. Measuring IgG in new-borns therefore reflects maternal exposure to pathogens. In cases of a chronic infection, reinfection or reactivation the production of IgG increases. Therefore, measuring the level of IgG can provide information about an on-going or resumed replication of the pathogen.

The association between schizophrenia and exposure to neurotropic pathogens, including herpes viruses and *Toxoplasma gondii*, has been investigated in several types of cohorts using different kinds of material (blood, cerebrospinal fluid (CSF) and post-mortem tissue) and techniques (PCR and pathogen-specific antibodies). These previous findings have been thoroughly reviewed elsewhere.^6,7,12–14^ Various studies measured the seroprevalence and levels of pathogen-specific IgG in serum and CSF in cross sectional cohorts to assess exposure and reactivity or reinfection of these pathogens during life. Some studies found spectacular increases in seroprevalence or IgG titers in patients with schizophrenia as compared with controls. However, other studies could not replicate these findings. Important confounders such as age, gender, ethnicity, and urbanicity were not always included in these studies, which could have contributed to inconsistent results.

This study aims to investigate whether prior exposure to a broad range of neurotropic pathogens, including HSV-1/HSV-2, varicella zoster virus (VZV), EBV, CMV and TG, is associated with schizophrenia and whether signs of increased rates of replication can be found in patients as compared to healthy controls. This study is one of the larger studies to date and it accounts for the most important confounders including age, gender, ethnicity, level of education, urbanicity at birth, and size of household.

## Materials and methods

### Study population

The Genetic Risk And Outcome of Psychosis (GROUP) study, started in 2006, is a large multicenter study in the Netherlands. Details of the study design have been described elsewhere.^15^ In short, patients with a schizophrenia spectrum disorder were recruited from mental health centers throughout the Netherlands. Healthy controls were recruited through mailings to random addresses in the catchment region. Inclusion criteria for patients were: fluent in Dutch and diagnosis of a schizophrenia spectrum disorder according to the Comprehensive Assessment of Symptoms and History (CASH)^16^ or Schedules for Clinical Assessment in Neuropsychiatry (SCAN)^17^ interview using the DSM-IV criteria. Eligible healthy controls had to meet the following criteria: fluent in Dutch, no history of a lifetime psychotic disorder or lithium use and no first- or second-degree family member with a lifetime psychotic disorder. At the second follow-up visit in 2009 plasma samples were collected from a subpopulation of the study and stored at −80 °C until further analysis. We included all available plasma samples from patients with a diagnosis of schizophrenia, schizophreniform disorder or a schizoaffective disorder at follow-up. The study was approved by the human ethics committees of the University Medical Centers of Utrecht, Amsterdam, Maastricht and Groningen. All included patients and healthy volunteers provided written informed consent before participating. Using questionnaires the highest level of completed education and size of the current household were assessed. Urbanicity of place of birth was assessed as previously described.^18^ This variable was dichotomized into being born in a low (0) or high urbanicity region (1). Data on current or previous comorbid physical disorders were collected through questionnaires.

### Measurement of IgG

IgG class antibodies against HSV-1, HSV-2, VZV, EBV, CMV, and TG were determined by commercial enzyme-linked immunosorbent assay (ELISA) tests (IBL Laboratories, Hamburg, Germany) according to the manufacturer’s protocols. Sensitivities and specificities of these tests are all >95%. In short, plasma samples were diluted and applied to 96-well microtiter plates that were precoated with pathogen-specific antigens. Patients and controls were equally distributed on the plates. After 1-h incubation and extensive washing, an enzyme-labeled anti-human IgG antibody was added for 30 min. Bound IgG was visualized by adding tetramethylbenzidine substrate, followed by adding H_2_SO_4_. The absorbance was measured at 450 nm using an ELISA microwell plate reader. A cut-off control sample was provided with the test for the qualitative interpretation of the results. Samples above the cut-off samples were scored positive, below the cut-off negative. IgG levels for CMV, HSV-1, and HSV-2 were quantified by calculating (patient absorbance value×10)/(absorbance value of the cut-off) from all seropositive subjects and expressed as units per ml. IgG levels for EBV, VZV, and TG were determined by using the calibration curve provided with the test and expressed as units per ml.

### Statistical analysis

Differences in baseline characteristics between the two diagnostic groups were examined using *χ*^2^ and Mann–Whitney *U-*tests when appropriate. To assess whether the prevalence of IgG antibodies against the six pathogens differed significantly between the group of patients and the control subjects *χ*^2^-tests were used. Titers of positive cases were compared between patients and controls using Mann–Whitney *U*-tests. A multiple logistic regression model was used to calculate odds ratios (ORs) for schizophrenia spectrum disorders in the groups testing positive for exposure to the various pathogens as compared to the groups testing negative. This model was adjusted for age, gender, ethnicity, level of education, urbanicity of place of birth, and size of household.

## Results

Plasma samples of 368 patients and 282 healthy controls were examined. An overview of the demographics and clinical characteristics of patients and controls is depicted in Table 1.

As shown in Table 2 no significant differences between patients and controls were found for HSV1 (*X*^*2*^=2.761 *P*=0.097), HSV2 (*X*^*2*^=0.406, *P*=0.524), EBV (*X*^*2*^=0.328, *P*=0.567) and TG (*X*^*2*^=0.060, *P*=0.806) seropositivity. A significantly higher seroprevalence of VZV (98.9 vs. 95.6%, *X*^*2*^=6.068, *P*<0.05) and CMV (40.4 vs. 27.7%, *X*^*2*^=11.621, *P*<0.001) was found in controls as compared to patients, without adjustment for possible confounders. The IgG titers of the positive cases did not differ significantly between patients and controls for any of the six pathogens.

The effects of adjusting for possible confounding factors are shown in Table 3. When applying the possible confounders separately the negative association with VZV and CMV remained significant. When applying the fully adjusted regression model no statistically significant association was found between seropositivity for any of the six pathogens and schizophrenia spectrum disorders.

## Discussion

In the present study, we did not find evidence for an increased exposure to HSV-1, HSV-2, EBV, and TG in plasma of patients with schizophrenia spectrum disorders as compared to healthy controls. We found a significantly higher seroprevalence of VZV and CMV IgG in controls as compared to patients. However, these differences did not remain statistically significant after correcting for multiple possible confounders. No differences in IgG titers were found between patients and controls for the seropositive cases.

A meta-analysis of previous findings on various infectious agents in schizophrenia did not find evidence for an increased exposure to HSV-1, VZV, EBV, and CMV in patients.^13^ Our results are in accordance with this meta-analysis. However, this meta-analysis did find a higher exposure to HSV-2 and TG in patients. We were unable to replicate these findings in our study. The authors of the meta-analysis mention that results on HSV-2 are strongly dependent on one large study that used blood samples obtained at birth and therefore assessed maternal IgG.^19^ This study design is incomparable to ours, which could explain the conflicting results.

In contrast to our results, numerous studies have found an association between schizophrenia and IgG type TG antibodies, described in several meta-analyses.^12–14,20^ Publication bias and the influence of confounders are most likely involved in this discrepancy. In 2012, Arias *et al.* described that studies that did not find an association between TG and schizophrenia seemed to have a more thorough design than those that do find this association.^13^ Since then, several larger studies were also unable to detect a difference between patients and controls in TG antibody levels^21–24^ or seropositivity.^24,25^ Moreover, Sutterland *et al* found signs of publication bias in his recent meta-analysis, although a significant positive association between TG and schizophrenia remained after correcting for this bias.^20^ Importantly, age, ethnicity, urbanicity, and social contact are associated with exposure to TG. In most studies age and gender were included as confounders, but the inclusion of the others was highly variable. In the present study we were able to control for age, gender, ethnicity, level of education, urbanicity at birth and size of household. In addition to this, contact with felines and consumption of raw meat are major risk factors for TG infection. A previous study that included these confounders found that they had a significant effect on the association between schizophrenia and TG.^26^ Unfortunately data on these confounders were not available in the present study, as well as most of the previous studies, and could therefore be involved in the inconsistent results.

Interestingly, we found a significant higher exposure to CMV in controls in our unadjusted model, which remained significant after adjusting for multiple possible confounders separately, but not after applying the fully adjusted model. This could be due to lack of statistical power. Two other recent studies also found a significantly higher exposure^27^ and titer^23^ of CMV in controls as compared to patients. These results seem contra-intuitive but could be explained by a decreased exposure to CMV during life due to the more isolated lifestyle of patients, a protective effect of CMV infection on the risk of developing schizophrenia or as sign of an altered immune function in schizophrenia.^23^ Some support for the latter hypothesis comes from a metagenomic analysis performed with the virus discovery method VIDISCA-454 that revealed a significantly lower viral prevalence in a group of pregnant mothers of offspring with schizophrenia. Consistent with the existing inverse correlation between the level of these viruses and the immunocompetence of an individual, Canuti *et al* hypothesized the presence of a higher immune activity during pregnancy in mothers whose offspring later develop a psychotic disorder as compared to controls.^28^ In addition to this, CMV is known for modulating MHC class I antigen presentation pathways^29^ and variations in the MHC region on chromosome 6p21.3–22.1 are highly associated schizophrenia.^3^

We were unable to retrieve other studies that also found an increased exposure rate to VZV in controls as compared to patients. However, only very little patients and controls tested negative for VZV, which makes it unlikely that the difference between patients and controls is relevant for our understanding of schizophrenia.

Strong aspects of our study include the large sample size and the correction for multiple relevant confounders. Our study also has several limitations. As described earlier we did not have data on all relevant confounders. Furthermore, this study is limited by its cross sectional design. Patients could indeed have had an increased exposure to the examined pathogens during prenatal^6^ or childhood^7^ periods, but these differences might not be detectable anymore in adulthood as exposure to these pathogens in later stages of life might be similar or higher in controls. Signs of increased replication of neurotropic pathogens could be state-specific and only detectable in acutely ill patients or patients with a first episode psychosis that are lacking in this study. Last, plasma levels of IgG do not necessarily reflect intrathecal production of antibodies and are therefore apt to underestimate brain immune responses against these pathogens.

In conclusion, we found no evidence to support the hypothesis that infection with HSV-1, HSV-2, VZV, EBV, CMV, and TG after birth has a role in the pathogenesis of schizophrenia. However, we emphasize that findings from prenatal cohorts, childhood cohorts before disease onset and adult patients should be clearly distinguished to further unravel the link between infectious agents and schizophrenia.

## Figures and Tables

**Table 1:** Demographics and clinical characteristics of patients and controls

	*Patients (*N*=368)*	*Controls (*N*=282)*	*Group comparison*
Mean age (s.d.) in years	30.5 (±7.0)	34.5 (±10.5)	*P*<0.01
Range	18–55	18–55	
Gender M/F (% males)	281/87 (76%)	118/164 (42%)	*P*<0.01
Diagnosis	Schizophrenia: 295		
	Schizoaffective disorder: 58		
	Schizophreniform disorder: 15		
Mean duration of illness in years	7.4 (±4.3)		
Range	2–43		
Currently using antipsychotics yes/no/NA	257/37/74		
Ethnicity Caucasian yes/no/NA	310/50/8	262/16/4	*P*<0.01
Completed selective secondary education or higher yes/no/NA	241/126/1	274/8/0	*P*<0.01
High urbanicity yes/no/NA	109/227/32	87/181/14	*P*=0.995
Single household yes/no/NA	148/167/53	57/202/23	*P*<0.01
Reported current or previous atopic, inflammatory or autoimmune disorder yes/no/NA	94/96/178	96/89/97	*P*=0.640

Abbreviations: F, female; M, male; NA, not available.

**Table 2:** Prevalence and titer of IgG antibodies against pathogens in patients and controls

	*Patients (*N*=368) positive/negative (% positive)*	*Controls (*N*=282) positive/negative (% positive)*	*Group comparison*	*Patients IgG level (median and IQR)*	*Controls IgG level (median and IQR)*	*Group comparison*
HSV-1	133/235 (36.1%)	120/162 (42.6%)	*P*=0.097	31.0, 19.2	33.3, 20.6	*P*=0.145
HSV-2	11/357 (3.0%)	11/271 (3.9%)	*P*=0.524	19.4, 4.9	30.9, 32.8	*P*=0.178
VZV	352/16 (95.6%)	279/3 (98.9%)	*P*<0.05	77.2, 142.6	83.2, 146.6	*P*=0.451
EBV	264/104 (71.7%)	208/74 (73.8%)	*P*=0.567	19.8, 6.3	19.9, 7.5	*P*=0.906
CMV	102/266 (27.7%)	114/168 (40.4%)	*P*<0.001	33.7, 16.0	34.5, 18.5	*P*=0.856
TG	68/300 (18.5%)	50/232 (17.7%)	*P*=0.806	227.8, 107.7	197.6, 102.9	*P*=0.232

Abbreviations: IgG, immunoglobulin G; IQR, interquartile range.

**Table 3:** Exposure to pathogens and risk of schizophrenia spectrum disorder

*Adjustment for confounding*	*Sample size*	*HSV1*	*HSV2*	*VZV*	*EBV*	*CMV*	*TG*
*Odds ratio (95% confidence interval)*							
Unadjusted	650	0.764 (0.556–1.050)	0.759 (0.324–1.777)	0.237 (0.068–0.820)*	0.903 (0.637–1.280)	0.565 (0.406–0.786)**	1.052 (0.703–1.574)
Age	650	0.885 (0.636–1.231)	1.136 (0.469–2.754)	0.229 (0.065–0.809)*	1.011 (0.706–1.447)	0.598 (0.426–0.838)**	1.318 (0.864–2.010)
Gender	650	0.839 (0.597–1.178)	1.403 (0.569–3.459)	0.205 (0.056–0.750)*	1.023 (0.704–1.485)	0.651 (0.458–0.925)*	0.979 (0.637–1.506)
Ethnicity	638	0.728 (0.526–1.009)	0.805 (0.342–1.892)	0.248 (0.071–0.870)*	0.877 (0.615–1.250)	0.509 (0.362–0.717)**	1.125 (0.744–1.702)
Educational level	649	0.701 (0.495–0.993)*	0.952 (0.392–2.311)	0.224 (0.062–0.807)*	0.968 (0.662–1.416)	0.618 (0.432–0.882)**	0.883 (0.564–1.382)
Urbanicity	604	0.736 (0.528–1.025)	0.883 (0.353–2.206)	0.260 (0.074–0.915)*	0.887 (0.618–1.273)	0.500 (0.354–0.706)**	0.967 (0.639–1.464)
Single household	574	0.822 (0.579–1.166)	0.705 (0.273–1.826)	0.265 (0.073–0.957)*	0.885 (0.605–1.296)	0.585 (0.406–0.845)**	0.862 (0.548–1.355)
Fully adjusted	524	0.773 (0.490–1.217)	2.160 (0.639–7.298)	0.265 (0.057–1.227)	1.128 (0.700–1.815)	0.663 (0.416–1.057)	0.992 (0.548–1.795)

Abbreviations: CMV, cytomegalovirus; EBV, Epstein–Barr virus; HSV, herpes simplex virus; TG, Toxoplasma gondii; VZV, varicella zoster virus.

*P<0.05; **P<0.01.
